# Improvement of the Measurement Range and Temperature Characteristics of a Load Sensor Using a Quartz Crystal Resonator with All Crystal Layer Components

**DOI:** 10.3390/s17051067

**Published:** 2017-05-08

**Authors:** Yuichi Murozaki, Shinya Sakuma, Fumihito Arai

**Affiliations:** Department of Micro-Nano Systems Engineering, Nagoya University, Nagoya 464-8603, Japan; sakuma@mech.nagoya-u.ac.jp (S.S.); arai@mech.nagoya-u.ac.jp (F.A.)

**Keywords:** quartz crystal resonator (QCR), biosignal detection, load sensor, wide-range

## Abstract

Monitoring multiple biosignals, such as heart rate, respiration cycle, and weight transitions, contributes to the health management of individuals. Specifically, it is possible to measure multiple biosignals using load information obtained through contact with the environment, such as a chair and bed, in daily use. A wide-range load sensor is essential since load information contains multiple biosignals with various load ranges. In this study, a load sensor is presented by using a quartz crystal resonator (QCR) with a wide measurement range of 1.5 × 10^6^ (0.4 mN to 600 N), and its temperature characteristic of load is improved to −7 Hz/°C (−18 mN/°C). In order to improve the measurement range of the load, a design method of this sensor is proposed by restraining the buckling of QCR and by using a thinner QCR. The proposed sensor allows a higher allowable load with high sensitivity. The load sensor mainly consists of three layers, namely a QCR layer and two holding layers. As opposed to the conventional holding layer composed of silicon, quartz crystal is utilized for the holding layers to improve the temperature characteristic of the load sensor. In the study, multiple biosignals, such as weight and pulse, are detected by using a fabricated sensor.

## 1. Introduction

Monitoring vital conditions, such as heart rate, respiration cycle, and weight transitions, plays an important role in daily life. Individuals can use the information for health management [1,2,3,4,5,6]. In order to support activities for health management based on such monitoring, it is important that a sensing method should not involve excessive efforts by individuals for the sensing of biosignals. Thus, sensing devices should be in total harmony with the living environment, such as rooms, clothes, and belongings, and should be capable of casually measuring biosignals with high precision in daily life. We call such methods “casual sensing” [7]. Sudden changes in vital conditions in daily life are detected by using casual sensing devices. Load signals include vital conditions, such as a heart beat, as well as physical conditions, such as signs of illness that affect the behavior of individuals. Therefore, it is possible to evaluate health conditions by measuring multiple biosignals using load sensors. Load signals from multiple biosignals correspond to various ranges and, thus, a wide measurement range is a key requirement for a load sensor. For example, a heartbeat is on the order of tens of milliNewtons, respiration is on the order of sub-Newtons, and body motion is on the order of hundreds of Newtons.

Several extant studies focused on biosignal detection using load sensors and included pulse detection using strain gauge-type sensors [8,9], pulse and respiration detection using piezoelectric-type sensors [10,11,12], and detection of body motion while sleeping [13] or during sports [14] using capacitance change-type sensors. Although these sensors can non-invasively measure vital information or body motions of individuals, the measurement range does not cover multiple biosignals such as a heart rate signal under a weight-loaded condition. Conversely, we have been developing load sensors using a QCR (QCR load sensor). Specifically, the characteristic of a QCR includes linearly changing the resonant frequency with the magnitude of the applied load [15,16]. A quartz crystal possesses high strength against compressive stress and, thus, a wide measurement range for load sensing is expected. We have succeeded in fabricating a miniaturized QCR load sensor with dimensions of 2 mm × 2 mm × 1.04 mm using MEMS techniques [17]. The sensor mainly consists of three layers, namely a QCR layer and two silicon (Si) layers. Additionally, multiple biosignals, such as body motion, respiration, and pulse signals, were successfully detected by integrating the sensor into a chair because the fabricated sensor possesses a wide measurement range. It is considered that load sensors with an expanded measurement range can detect weight, as well as pulse, signals by setting the sensors in the floor or weighing scales. However, there is a paucity of extant research that focuses on improvements in the measurement range. It is necessary to design a sensor by considering the destruction limit of a sensor to expand the measurement range. Furthermore, a QCR load sensor involves a problem related to the temperature characteristics due to a mismatch between the temperature coefficients of expansion of the quartz crystal and silicon [7,17,18]. In practical use situations, the stability of sensor output is significantly affected by the environmental temperature fluctuation. Thus, improvements in temperature characteristics are also necessary to achieve a wide-range of measurements in practical use.

In this study, a design method of the QCR load sensor is proposed to expand its measurement range by restraining the buckling of the QCR and also by using a thinner QCR. The fundamental characteristics of the fabricated sensor are evaluated. The results indicate that a measurement range of 1.5 × 10^6^ is achieved. Additionally, an all-quartz crystal structure is proposed for the sensor in which the QCR load sensor is composed of all crystal layer components. The sensor consists of a QCR layer and two holding layers. The base material of the three layers is completely composed of quartz crystal. In a previous study, the two holding layers were composed of silicon [7]. The usage of quartz crystal holding layers significantly reduces the sensor output fluctuation caused by temperature. Finally, measurements of weight and pulse are performed to confirm the effectiveness of the fabricated sensor. The results indicated that multiple biosignals of pulse and weight are successfully detected.

## 2. Measurement Range of QCR Load Sensors

AT-cut QCRs possess superior temperature stability at room temperature. AT-cut QCRs generate thickness-shear vibration when an oscillation circuit is connected, and the frequency change is obtained depending on the applied force. The frequency change (Δf) is proportionally related to the applied force (P):

(1) $$\Delta f=S_{s}P$$

where $S_{s}$ denotes sensor sensitivity given by the following expression:

(2) $$S_{S}=\beta \frac{\eta }{wt^{2}}$$

where β denotes the coefficient of sensitivity determined by direction of the applied force, η denotes the load transfer efficiency, and w and t denote the width and thickness of the QCR, respectively. We defined load transfer efficiency as the ratio of the compressive load on the QCR to the vertical component of the external force. Equation (2) indicates that a reduction in the thickness of the QCR is efficient in improving sensor sensitivity.

The practical resolution of load $P_{res}$ is defined as follows:

(3) $$P_{res}=\frac{f_{F}}{S_{S}}$$

where $f_{F}$ denotes fluctuation of the sensor output that indicates stability of the oscillation frequency. Conversely, the theoretical maximum allowable load $P_{max}$ is given as:

(4) $$P_{max}=\frac{\sigma_{max}\cdot wt}{\eta }$$

where $\sigma_{max}$ denotes the allowable stress of AT-cut quartz crystals. Equation (4) indicates that the maximum measurable load improves with decreases in the load transfer efficiency. The theoretical measurement range (ρ) corresponds to the ratio of the maximum allowable load to the practical resolution and is given by Equations (3) and (4) as follows:

(5) $$\rho =\frac{P_{max}}{P_{res}}=\frac{\beta \cdot \sigma_{max}}{f_{F}t}$$

The aforementioned proportional relation indicated that the factors necessary to achieve a wide-range QCR load sensor include decreasing the QCR thickness, increasing the stability of the sensor output, and enhancing the maximum allowable load.

It is necessary to consider the following points: The resonant frequency of the QCR increases with decreases in the QCR thickness. That is, the resonant frequency of the QCR is inversely related to thinning of the QCR thickness:

(6) $$f\;=\frac{1.67}{t\;}$$

where f denotes the resonant frequency of QCR in an infinite plane plate and t denotes the thickness the QCR in mm. The stability of the sensor output is related to the *Q* factor of the QCR. A high *Q* factor of the QCR is necessary to obtain a highly stable output. The maximum value of the *Q* factor obtainable for AT-cut crystal units at ordinary temperatures corresponds to an inverse function of frequency. The results indicate that the values of the *Q* factor range from 15 × 10^6^ at 1 MHz to 0.15 × 10^6^ at 100 MHz [19]. The *Q* factor is given by Equation (7):

(7) $$Q=\frac{15\times 10^{7}}{f\;}$$

The theoretical maximum value of *Q* factor decreases with reducing the QCR thickness. Thus, with respect to reducing the QCR thickness, it is necessary to consider the electrode thickness, electrode diameter, shape of the QCR, and wiring of the QCR to ensure a high value of the *Q* factor.

**Figure 1:**
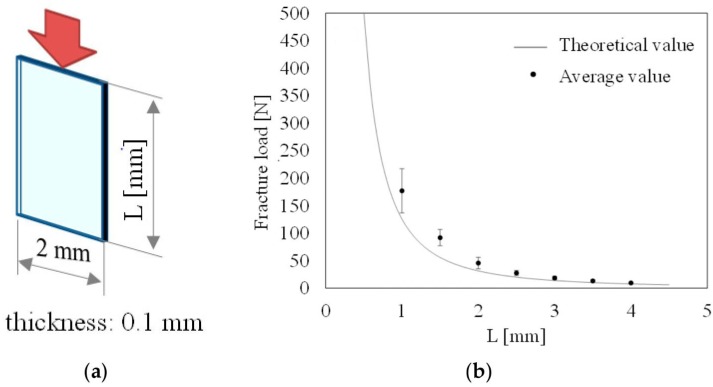
Failure stress of the quartz crystal chips: (**a**) Dimensions of the test piece; the (**b**) experimental result and theoretical value of Euler’s critical load.

The maximum allowable load depends on the buckling load of the QCR due to its thin structure. A failure test is conducted to examine the relationship between the failure stress and buckling load as determined by the structure of the QCRs. In the experiment, test pieces of quartz crystal with different lengths (100 μm thickness, 2 mm width, lengths ranging from 1 mm to 4 mm, in 0.5 mm increments) are used. The test pieces of quartz crystal are set on the load cell and compressed until they break down. The applied load at failure is measured by a load cell (9031A, Kistler Corporation, Winterthur, Switzerland). The average value of the failure load of each shape of test pieces is shown in Figure 1. The error bars indicate standard deviation. Specifically, 20 test pieces are used for each size in the experiment. The theoretical value expresses Euler’s critical load for hinged ends as expressed by Equation (8):

(8) $$P_{cr}=\frac{\pi^{2}EI}{l^{2}}$$

where E denotes the modulus of elasticity, I denotes the area moment of inertia of the cross-section of the test piece, and l denotes the unsupported length of the column.

The results indicate that the shorter test piece is associated with higher failure stress, and this is considered to depend on the buckling stress. Furthermore, a test piece with a length of 1 mm indicated a failure stress of 900 MPa (when converted from the failure load of 180 N divided by the cross-section, which corresponded to an area of 0.2 mm^2^). The failure stress corresponds to six times the general tensile strength of 150 MPa. The quartz crystal consists of a crystalline material with low dislocations. The low dislocation results in the increased mechanical stability of quartz [20]. Consequently, a quartz crystal possesses high strength relative to the compressive stress and is fragile relative to the tensile or bend stress. The result suggests that preventing the buckling of the QCR leads to an increase in the allowable stress of the QCR load sensor.

## 3. Design of the QCR Load Sensor

**Figure 2:**
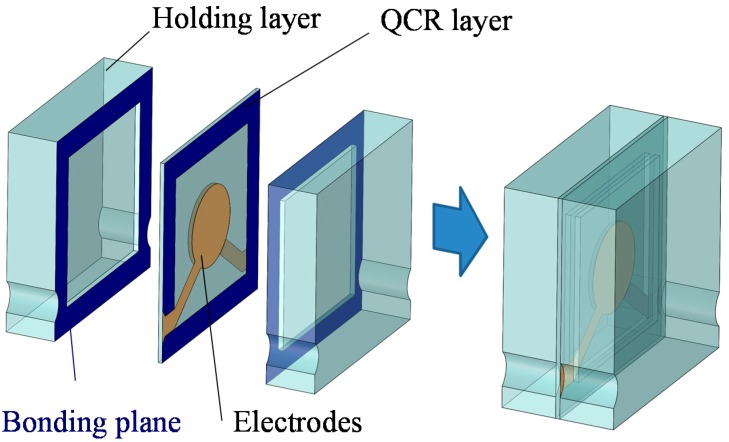
Fracture stress of the quartz crystal chips.

A design of the QCR load sensor is proposed to miniaturize and enlarge the measurement range. The sensor consists of three layers, namely two holding layers (with a thickness of 500 μm) and a QCR layer as shown in Figure 2. The thickness of the QCR is reduced when compared with that of a conventional sensor [18] to improve the measurement range of the QCR load sensor. Etching processes of quartz crystals can reduce the QCR thickness. However, the etching processes deteriorates the surface roughness and flatness, which leads to a low *Q* of the QCRs. Thus, a quartz crystal wafer with a thickness of 41.7 μm and double-sided mirror-polished surface is used. It is necessary to ensure that the diameter of the electrodes is 15–20 times the QCRs thickness in order to obtain a stable output of the QCRs [20]. Thus, the diameter of the electrodes is determined as 0.8 mm. Additionally, it is necessary for the outer shape of the QCR to be sufficiently high when compared with the diameter of the electrodes. Thus, the outer size of the QCR load sensor is determined as 2 mm × 2 mm. Miniaturization of the sensor can also be achieved by thinning down the QCR. The QCR layer is firmly fixed by the two holding layers bonded at the four corners. This structure enables the QCR sensor to withstand breakage due to bending and buckling. Furthermore, the applied load is shared by the holding layers and it can realize a high maximum allowable load. The surface of each holding layer is slightly etched to avoid interference with the oscillation. A wiring connection is placed on the bonding part to consider the effect of oscillation. Thus, connection holes are designed for the wiring at the holding layers. However, these holes could involve defects due to stress concentration. Consequently, the arc-shaped holes are fabricated to reduce stress concentration. Quartz crystal is used for the substrate of the holding layers to prevent sensor output fluctuation caused by temperature due to thermal stress generated by the difference in thermal expansion between the QCR and the holding layers.

**Figure 3:**
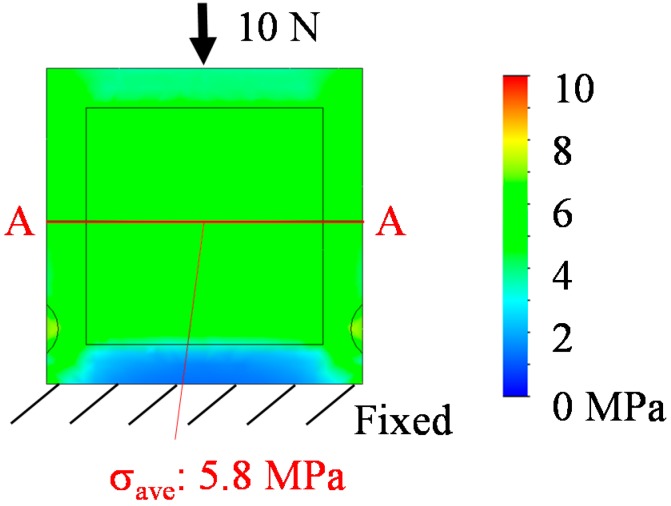
Results of the FEM analysis.

Analysis of the buckling load of the proposed sensor structure is evaluated using finite element method (FEM) analysis with SolidWorks Simulation (2014 SP5.0, SolidWorks Corp., Waltham, MA, USA). As a result, the buckling load of the QCR layer corresponds to 996 N (stress at center of QCR layer was calculated as 573 MPa). Conversely, the buckling load of the quartz crystal layer component with 41.7 μm thickness and 2 mm outer size corresponds to 4.5 N (54 MPa). The proposed sensor structure can improve the buckling load such that it is ten times higher than that of the QCR single layer. Additionally, load transfer efficiency is calculated using FEM analysis with SolidWorks Simulation. Von Mises stress of the sensor is analyzed with the assumption that a perpendicular compressive load of 10 N is applied to the sensor tip. The analytical results are shown in Figure 3. The results indicate that the load applied to the QCR corresponds to 0.48 N (it is calculated as 5.8 MPa by multiplying the average stress on the A-A cross-section with an area of 0.083 mm^2^). In the analysis, the load transfer efficiency is calculated as 4.8%. The low load transfer efficiency can be used to obtain a high maximum allowable load of the sensor.

## 4. Fabrication and Evaluation

### 4.1. Fabrication

**Figure 4:**
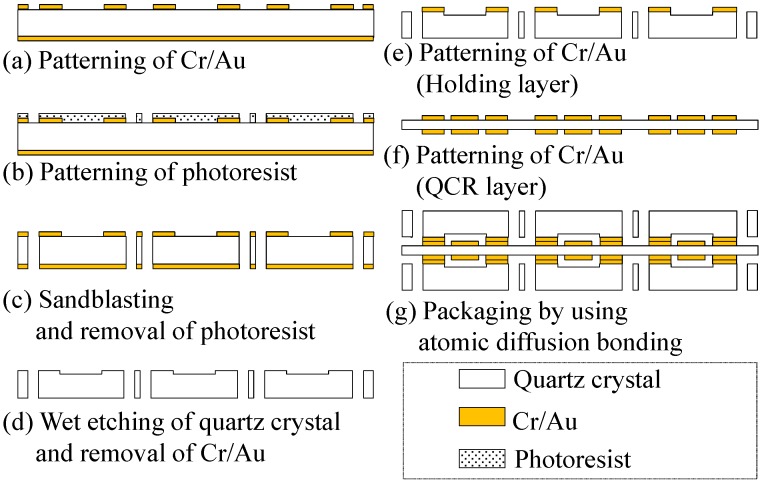
Process flow of the sensor fabrication.

The sensor is fabricated with the following procedure that uses microfabrication techniques. A Cr/Au layer is patterned as the mask for wet etching of a quartz crystal substrate with a thickness of 500 µm. The opposite side is fully covered by Cr/Au to avoid wet etching (Figure 4a). A photoresist is patterned on the quartz crystal substrate. The resist pattern is used as the mask for sandblasting (Figure 4b). The quartz crystal substrate is etched by using a sandblasting technique to form a hole for wiring (Figure 4c). The quartz crystal layer is etched by a wet etching technique. The depth of clearance is set as 10 µm (Figure 4d). The Cr/Au layer is sputtered on the quartz crystal substrate. The Cr/Au layer is used as a bonding material between the QCR layer and the holding layer for the atomic diffusion bonding process. The Cr/Au is patterned on both surfaces of the substrate, and the pattern is used as the electrode of the QCR and the bonding material (Figure 4e,f). The atomic diffusion bonding technique is used to bond the QCR layer and the two holding layers (Figure 4g). The packaged sensor array is then diced, and wiring is attached using a conductive silver paste.

**Figure 5:**
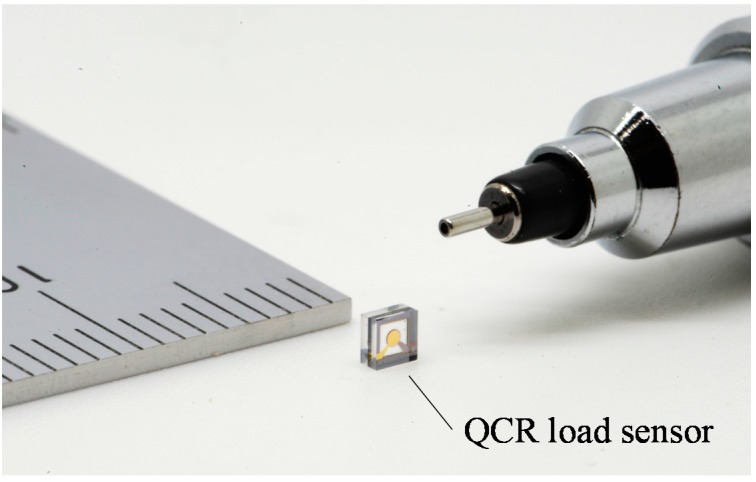
Fabricated QCR load sensor.

Figure 5 shows a photograph of a fabricated sensor. The sensor size corresponds to 2 mm width, 2 mm height, and 1.04 mm thickness. The impedance characteristic is measured by an impedance analyzer (ZA5405, NF Corp., Yokohama, Japan) to evaluate the resonance characteristics of the QCR load sensor. The measurement results reveal that the equivalent circuit constants are as follows: a resonance frequency (*f*) of 37.881 MHz, equivalent electrical resistance (R_1_) of 32.6 Ω, and an inductance (L_1_) of 9.55 mH. The *Q* of the fabricated sensor is calculated as 6.9 × 10^4^. The value of *Q* is significantly low when compared with the theoretical maximum *Q* value that corresponds to 3.7 × 10^5^ as calculated by Equation (7).

### 4.2. Loading Characteristic

**Figure 6:**
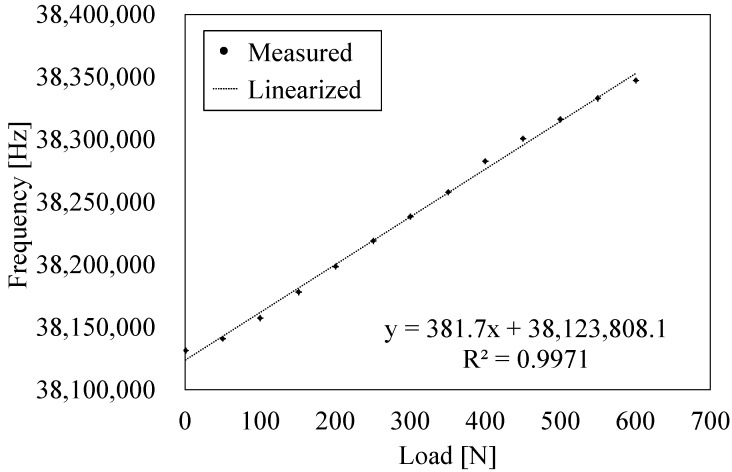
Results of the loading test.

The fabricated load sensor is calibrated by a loading test. In the experiment, an oscillation circuit and the sensor are connected, and the sensor output is measured by a frequency counter (53132A, Agilent Technologies, Santa Clara, CA, USA). The QCR load sensor is fixed on the load cell (9031A, Kistler Corporation, Winterthur, Switzerland) and it is fixed on the Z-stage. The applied load is changed from 0 N with 20 N increments in steps until it breaks. The measured results and the linear fitting results are shown in Figure 6. The linear fitting line is expressed as follows:

(9) Y= ax + b

where *a* = 381.7 Hz/N denotes the sensitivity of the QCR load sensor, and *b* = 38,123,808.1 Hz denotes the unloaded measured frequency. The square of the correlation coefficient between the experimental results and the linear fitting line corresponds to R^2^ = 0.997. The applied load *x* N can be calculated by the oscillating frequency *Y* MHz using Equation (9). It is confirmed that the fabricated sensor can withstand a maximum load of 600 N. Accordingly, load transfer efficiency is used to calculate the failure stress at the QCR layer as 345 MPa. The fracture stress exceeds 150 MPa, which is conventionally used as a failure stress. As a result, the maximum allowable load is successfully improved by the proposed sensor structure. Conversely, the value is lower when compared with the result of the analytical calculation of buckling stress (573 MPa). The fracture of the sensor occurs from the bonding plane, and it is expected that the allowable stress increases with increases in the bonding condition.

### 4.3. Stability of Sensor Output

**Figure 7:**
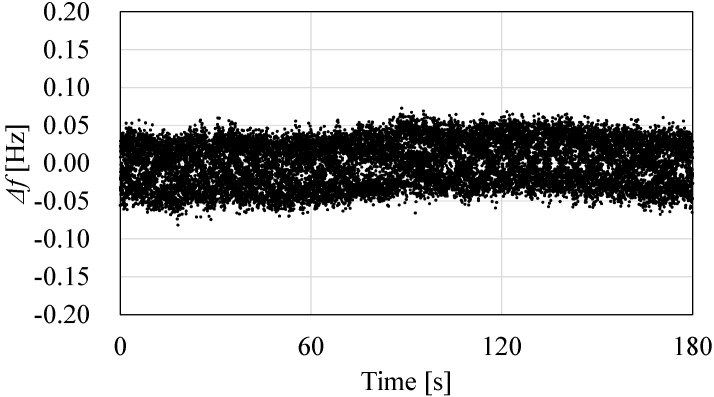
Sensor output for 3 min, where the maximum value, minimum value, and standard deviation are 0.07–0.08 Hz, and 0.03 Hz, respectively.

The time stability of the sensor output is measured with a sampling frequency of 100 Hz. In the experiment, an oscillation circuit and the sensor are connected, and the sensor output is measured with a frequency counter (53132A, Agilent Technologies Santa Clara, CA, USA). The sensor is set in an adiabatic condition. The measurement is conducted after the sensor output reaches a steady state. Figure 7 shows the result of the sensor output for 3 min in a stable condition. The vertical axis shows the differences in frequency (Δ*f*) between the measured frequency and average value of the measured frequency for 3 min. From the result, the stability that corresponds to the frequency fluctuation of the sensor output is measured as 0.15 Hz, which is the difference between the maximum value and the minimum value. The practical resolution is calculated as 0.4 mN from the fluctuation and sensor sensitivity.

### 4.4. Temperature Characteristics

**Figure 8:**
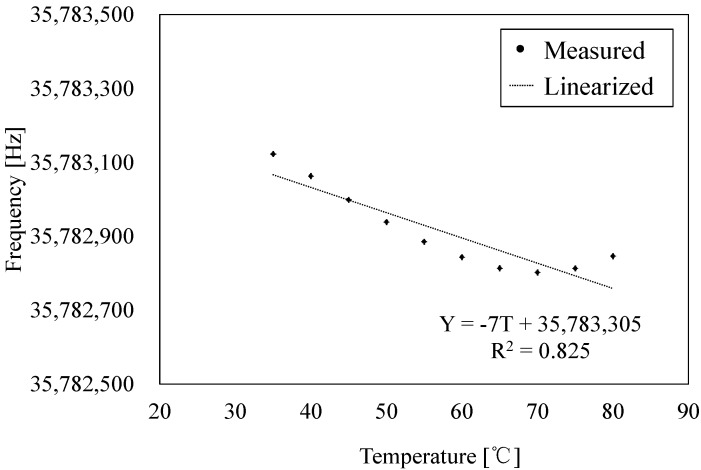
Temperature characteristic of the fabricated sensor.

Temperature characteristics of the fabricated sensors are evaluated by measuring the changes of the frequency output relative to the changes in temperature. In the experiment, the sensor connected to the oscillation circuit is set in the thermostatic chamber. The temperature in the thermostatic chamber is measured by thermocouples placed near the QCR load sensor. In the measurement, the temperature in the thermostatic chamber is gradually cooled from 80 °C to 30 °C, and the resonant frequency is measured with stepwise increments of 5 °C. Figure 8 shows the evaluation results of the temperature characteristics. The linear fitting was processed to calculate temperature sensitivity. The temperature sensitivity is calculated as −7 Hz/°C, which is defined as the slope of the linear fitting line, and it corresponds to −18 mN/°C. A quartz crystal is used for the substrate of the holding layers to prevent deterioration of temperature characteristics by thermal stress. The frequency change due to thermal stress is linearly proportional to the temperature change. It is observed that the resonant frequency gradually decreases in the range from 30 °C to 70 °C, and subsequently increases. It is considered that this temperature characteristic of the QCR load sensor is due to the characteristic of the AT-cut quartz crystal itself [21]. The frequency change due to thermal stress is barely observed to occur. Temperature compensation of the AT-cut QCR is easily performed by using a thermistor [22] or a differential method [7].

## 5. Application

**Figure 9:**
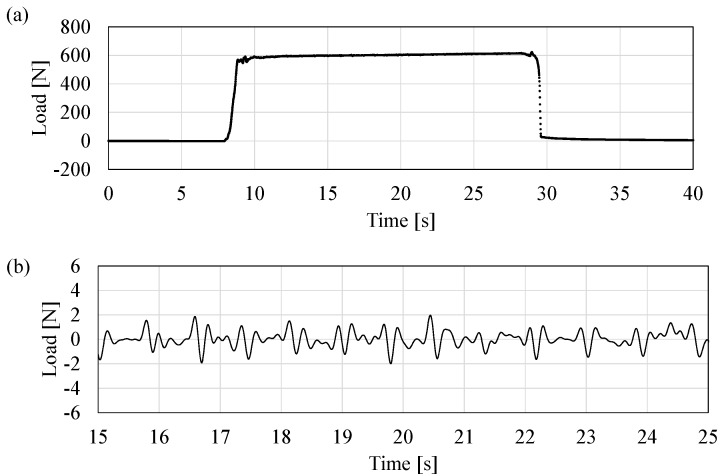
(**a**) Raw data; (**b**) band-pass filtered data of casual sensing of weight.

**Figure 10:**
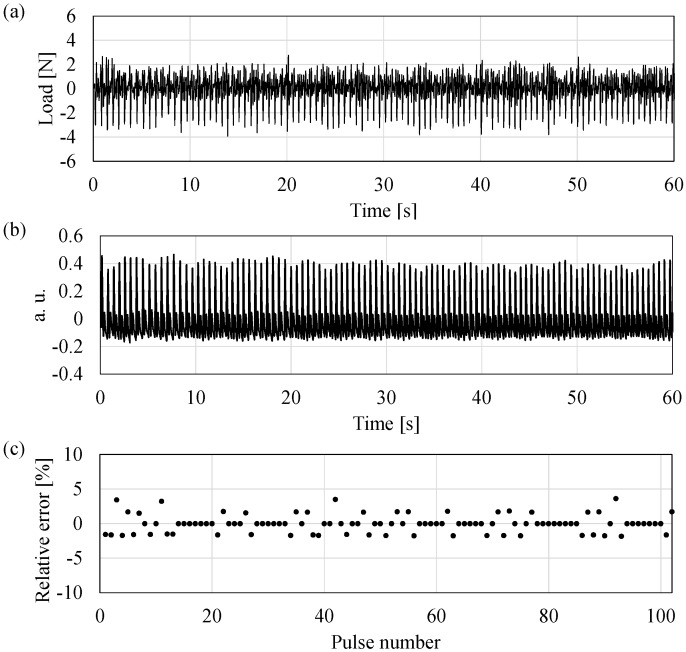
The results of the comparison between the developed load sensing system and the commercial available pulse wave meter: (**a**) band-pass filtered data (0.8–20 Hz) of the developed system; (**b**) band-pass filtered data (0.8–20 Hz) of the pulse wave meter; (**c**) the relative error with respect to the pulse number.

A multiple biosignal detection of weight and pulse is conducted to evaluate the effectiveness of the fabricated sensor. In the experiment, four sensors are fixed to the corners of a board. The applied load on the board is completely shared between the four sensors. The experimental result is shown in Figure 9a. The experimental procedure is as follows. A male subject aged 25 years, 62 kg in weight, and a height of 182 cm rides on the board 10 s after the measurement commences. The subject gets off the board after approximately 30 s. Additionally, 610 N of the load is measured after the subject rides on the board. Figure 9b shows the data after applying a band-pass filter ranging between 0.6 Hz and 20 Hz. A periodic change occurs when the subject rides on the board. A comparative experiment is conducted to estimate the pulse rate detection of the weighing scale using a QCR load sensor. In the experiment, a finger cuff-type pulse wave meter using an infrared sensor (Finapres Medical Systems, Finapres NOVA, Enschede, The Netherlands) is used as the reference of the pulse rate. The measurement commenced after the subject rides on the weighing scale. The subject stands upright without moving during the measurement. Figure 10a,b shows the data after applying a band-pass filter ranging between 0.8 Hz and 20 Hz. This is followed by comparing the peak-to-peak interval of the pulse rate results for approximately 1 min (102 peaks of pulse) in the cases of the sensor $t_{QCR}$ and the pulse wave meter $t_{\mathrm{ref}}$. Band-pass filtering and peak detection are conducted by using MATLAB R2012a (Mathworks Inc., Natick, MA, USA). Figure 10c shows the relative errors for each interval in which the relative error is defined as follows:

(10) $$Relative\;Error=\frac{t_{QCR}-t_{ref}}{t_{ref}}$$

The average error for 3 min corresponds to −0.01% and the standard deviation corresponds to 1.26%. From these results, it is confirmed that the fabricated sensor can simultaneously measure the weight and pulse information.

## 6. Discussion

### 6.1. Further Improvement of the Measurement Range

In this study, the achieved measurement range of the fabricated QCR load sensor corresponds to 1.5 × 10^6^. However, the fracture strength of the fabricated sensor is lower than the theoretical value based on the buckling stress analysis. It is considered that the fracture of the sensor occurs from the bonding surface. The results suggest that the maximum allowable stress of the sensor can be increased by improving the bonding strength. Additionally, with respect to the stability of the sensor output, the *Q* factor of the fabricated sensor is significantly less than the theoretical value calculated from the internal loss. It is expected that further improvement of the *Q* factor can be accomplished by optimizing the electrode shape of the QCR and sealing the QCR layer with a vacuum. As a result, the stability of the sensor output can be improved. In addition to the improvement instability, the measurement resolution can be improved. Further expansion of the measurement range is expected through these improvements. Enlarging the measurement range can achieve the biosignal detection of the pulse and walking steps by installing a wide-range load sensor under the floor.

### 6.2. Temperature Characteristics

**Figure 11:**
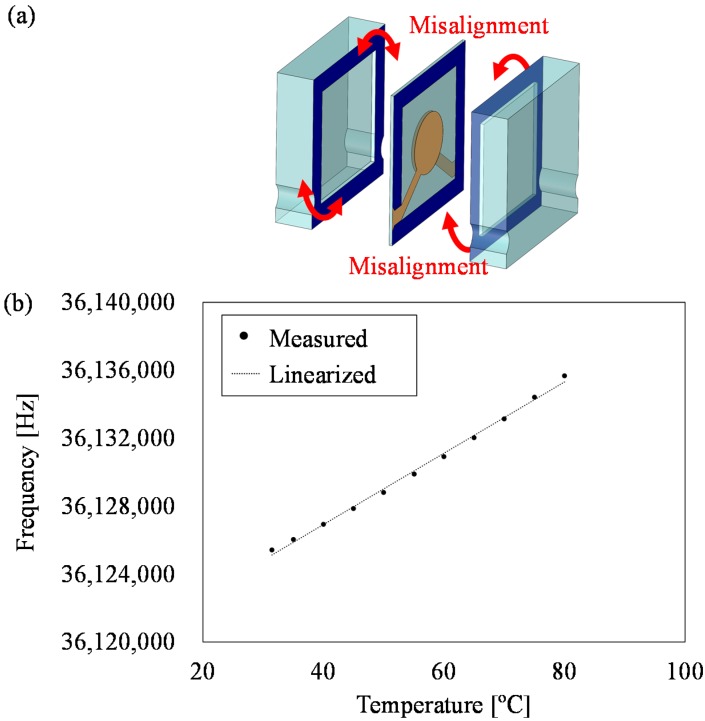
The influence of the misalignment of the bonding process: (**a**) a schematic image of the misalignment of bonding; (**b**) the temperature characteristics of the misaligned QCR load sensor. The temperature sensitivity corresponds to 210 Hz/°C.

In the study, quartz crystal is utilized as the material of the holding layers to obtain a stable temperature characteristic. In the fabrication of the QCR load sensor, it is important to consider the crystal orientation of the quartz crystal wafer. Quartz crystal is an anisotropic material, and the temperature coefficients of expansion vary based on the orientation. Therefore, there are two important keys for the fabrication. One key involves the cut direction of the quartz crystal substrate. The coefficient of thermal expansion differs with differences in the cut direction and, thus, it is necessary to use a quartz crystal substrate with the same cut direction as the QCR layer and holding layers. Therefore, a quartz crystal substrate with same cut direction is used in this study. The other key relates to the accuracy of alignment of quartz crystal substrates, as shown in Figure 11a. Angular misalignment causes the generation of thermal stress due to the differences in the coefficients of temperature expansion in various directions. An example of the misalignment is shown in Figure 11b. Figure 11b shows the temperature characteristic of all crystal load sensors with an angular misalignment of 0.6°. The accuracy of alignment of the fabricated sensor is calculated from the micro-photograph. The linear fitting was processed to calculate the temperature sensitivity. From the result, it is noted that the temperature sensitivity of the misaligned QCR load sensor corresponds to 210 Hz/°C, which corresponds to the slope of the linear fitting line. The frequency fluctuations caused by thermal stress have a linear relationship against temperature fluctuation. The temperature characteristic is largely influenced by thermal expansion caused by misalignment. Conversely, the angular misalignment of the full crystal sensor shown in Figure 8 is less than 0.02°, and the temperature characteristics have a nonlinear change. The temperature characteristic of the AT cut quartz resonator itself can be expressed in a cubic curve. Temperature characteristics of Figure 8 shows the temperature characteristics of the quartz oscillator itself, and it can be said that the influence of thermal stress is small. Consequently, the temperature sensitivity corresponds to −7 Hz/°C. Therefore, the accuracy of the angular alignment is important to obtain a stable temperature characteristic.

## 7. Conclusions

In this study, a design method of a QCR load sensor is proposed to improve the measurement range and its temperature characteristics. An all-quartz crystal structure is proposed for this sensor. That is, the QCR load sensor is composed of all crystal layer components. The thinner QCR layer is firmly fixed by two holding layers composed of crystal, and the four corners are bonded to prevent buckling. The proposed sensor structure can realize miniaturization, as well as high durability to perform loading with high sensitivity. It also enables the QCR load sensor to obtain a higher maximum allowable load by restraining the buckling of the thin QCR. As a result, the size of the fabricated sensor corresponds to 2 mm × 2 mm × 1.04 mm and with a measurement range of 4.0 × 10^−4^ N to 600 N (that is, a measurement range of 1.5 × 10^6^). Moreover, the temperature sensitivity improved to −7 Hz/°C (−18 mN/°C) by using all crystal layer components and focusing attention on the assembling accuracy of the components. Compared to previous sensors [19], the fluctuation of the sensor output is improved with the improvement of the temperature characteristics. Consequently, the resolution of the sensor is improved and the measurement range is expanded.
